# Real-world pharmacovigilance analysis and cross-database consistency assessment of resmetirom-related adverse events using FAERS and VigiAccess

**DOI:** 10.3389/fphar.2026.1898840

**Published:** 2026-10-09

**Authors:** Yali Zhong, Cong Hou, Lingzuo Zhao, Xiaobin Luo

**Affiliations:** 1 Department of Gastroenterology, Zigong First People’s Hospital, Zigong, China; 2 Department of Neurosurgery, Zigong Fourth People’s Hospital, Zigong, China

**Keywords:** adverse events, FAERS, pharmacovigilance, real-world study, resmetirom, signal detection, VigiAccess

## Abstract

**Objective:**

This study aimed to characterize the post-marketing safety profile and potential adverse event signals of resmetirom using the FAERS and VigiAccess databases.

**Methods:**

FAERS reports from the quarter of initial United States approval through 2026Q1 were analyzed. After deduplication, DEMO, DRUG, REAC, THER, and OUTC tables were integrated, and reports listing resmetirom as the primary suspect drug were included. Disproportionality analysis used four methods: reporting odds ratio (ROR), proportional reporting ratio (PRR), Bayesian confidence propagation neural network (BCPNN), and multi-item gamma Poisson shrinker (MGPS), with IC and EBGM as the respective Bayesian signal metrics. PTs meeting all four predefined criteria were defined as robust signals. Additional analyses assessed SOC/PT profiles, label expectedness, seriousness and outcomes, time-to-onset (TTO), Weibull patterns, and cross-database consistency with VigiAccess.

**Results:**

We identified 1,409 resmetirom-related FAERS reports and 1,417 VigiAccess reports. Sixty-two robust PT signals across nine SOCs were detected, mainly involving gastrointestinal disorders, investigations, hepatobiliary disorders, and skin and subcutaneous tissue disorders. Prominent signals included diarrhoea, nausea, pruritus, alanine aminotransferase increased, aspartate aminotransferase increased, abdominal pain upper, dizziness, vomiting, weight decreased, and hepatic cirrhosis. Among FAERS reports, 419 (29.7%) were serious and 65 (4.6%) reported death. Sixteen robust PTs were not listed as corresponding adverse reactions in either the current United States. Prescribing Information or European SmPC. Complete temporal information was available for 170 reports (12.1%); median TTO was 26 days (IQR, 5–66), and the Weibull shape parameter was 0.72 (95% CI, 0.64–0.80), indicating an early-failure pattern. SOC-stratified analyses showed heterogeneous onset patterns. FAERS and VigiAccess showed high descriptive concordance in SOC- and PT-level reporting patterns (Spearman ρ = 0.998).

**Conclusion:**

Resmetirom-related reports were mainly gastrointestinal, laboratory, hepatobiliary, and skin-related. Hepatobiliary signals warrant particular attention, while cross-database concordance provides supportive, non-causal pharmacovigilance evidence.

## 1 Introduction

Metabolic dysfunction-associated steatohepatitis (MASH), formerly referred to as nonalcoholic steatohepatitis (NASH), is a progressive form of metabolic dysfunction-associated steatotic liver disease characterized by hepatocellular steatosis, inflammation, hepatocellular injury, and varying degrees of fibrosis (Rinella et al., 2023a; Rinella et al., 2023b). With the increasing prevalence of obesity, type 2 diabetes mellitus, dyslipidemia, and other cardiometabolic disorders, the global burden of MASH continues to rise. Progressive MASH is an important cause of cirrhosis, hepatocellular carcinoma, end-stage liver disease, and liver transplantation (Rinella et al., 2023b; Sanyal et al., 2021). Its pathogenesis involves multiple interacting mechanisms, including insulin resistance, lipotoxicity, mitochondrial dysfunction, immune-inflammatory activation, and profibrotic signaling. This biological heterogeneity has contributed to substantial challenges in drug development. For many years, management of patients with moderate-to-advanced fibrosis relied primarily on lifestyle modification, weight reduction, and control of metabolic risk factors (Rinella et al., 2023b; Sanyal et al., 2021).

In 2024, resmetirom, a liver-directed and selective thyroid hormone receptor-β (THR-β) agonist, received accelerated approval from the United States. Food and Drug Administration (FDA) for adults with noncirrhotic NASH/MASH and moderate-to-advanced liver fibrosis, in conjunction with diet and exercise (United States. Food and Drug Administration). Resmetirom activates hepatic THR-β signaling, enhances fatty acid β-oxidation, improves lipid metabolism, and reduces intrahepatic fat accumulation, with downstream effects on inflammatory and fibrotic processes (Kannt et al., 2021; Harrison et al., 2021). In the pivotal phase III MAESTRO-NASH trial, resmetirom significantly improved both MASH resolution and liver fibrosis compared with placebo, providing important evidence supporting its clinical use (Harrison et al., 2024). The MAESTRO-NAFLD-1 trial further demonstrated reductions in liver fat and improvements in metabolic parameters with an overall acceptable tolerability profile (Harrison et al., 2023). However, randomized trials are conducted under controlled conditions with predefined eligibility criteria, standardized follow-up, and structured medication management. They therefore may not fully capture the safety profile of resmetirom in broader real-world populations.

This issue is particularly relevant in MASH because affected patients commonly have obesity, diabetes, dyslipidemia, cardiovascular risk factors, and multiple concomitant medications (Rinella et al., 2023b). Statins, glucose-lowering therapies, antiplatelet agents, and other cardiometabolic drugs are frequently co-administered, potentially complicating the interpretation of gastrointestinal symptoms, hepatobiliary abnormalities, laboratory changes, and drug-drug interactions. Current regulatory product information for resmetirom highlights clinically important safety concerns, including hepatotoxicity, gallbladder-related adverse reactions, and clinically relevant drug–drug interactions (United States. Food and Drug Administration). Moreover, as a recently approved innovative therapy, large-scale, long-term, real-world safety data on resmetirom remain limited. Therefore, post-marketing pharmacovigilance analyses based on spontaneous reporting systems are valuable for early detection of potential safety signals, supplementing real-world evidence beyond clinical trials, and supporting clinical risk management (Bate and Evans, 2009).

Accordingly, this study integrated data from the FDA Adverse Event Reporting System (FAERS) and VigiAccess to characterize the post-marketing safety profile of resmetirom. Four complementary disproportionality signal-detection methods were applied: the reporting odds ratio (ROR), proportional reporting ratio (PRR), bayesian confidence propagation neural network (BCPNN), and multi-item gamma poisson shrinker (MGPS). The information component (IC) and empirical bayes geometric mean (EBGM) were used as the corresponding signal metrics for BCPNN and MGPS, respectively. Adverse-event signals were further characterized at the system organ class (SOC) and preferred term (PT) levels, along with assessments of label expectedness, report seriousness, and clinical outcomes; time-to-onset patterns; Weibull distribution characteristics; and cross-database consistency between FAERS and VigiAccess. This study aimed to identify clinically relevant post-marketing reporting signals, characterize their temporal and seriousness profiles, and provide evidence to support risk recognition and safety monitoring during resmetirom treatment.

## 2 Materials and methods

### 2.1 Study design and data sources

This retrospective pharmacovigilance study used spontaneous reporting data from the United States. Food and Drug Administration Adverse Event Reporting System (FAERS) as the primary data source, with VigiAccess used for cross-database descriptive comparison and consistency assessment. FAERS data from 2024Q1, the quarter in which resmetirom received its initial United States. approval, through 2026Q1 were included. The analysis was initiated from the approval quarter to capture the earliest phase of post-marketing reporting while preserving the quarterly structure of the publicly released FAERS data files. Earlier quarters were not included because routine clinical exposure to resmetirom was not expected before regulatory authorization. The same 2024Q1–2026Q1 observation window was applied to both resmetirom reports and the background FAERS reference population to reduce temporal heterogeneity in reporting practices and background adverse-event distributions.

Quarterly FAERS data files were downloaded, and the DEMO, DRUG, REAC, THER, and OUTC tables were extracted. These tables were linked using PRIMARYID and CASEID to integrate demographic information, drug exposure, adverse-event records, treatment timing, and clinical outcomes. VigiAccess, which provides publicly accessible, aggregated data from the World Health Organization’s global individual case safety report database, was used as an external reference for descriptive cross-database comparisons (Huang et al., 2024). Because VigiAccess provides aggregated rather than individual-level case data, patient-level modeling, adjustment for confounding factors, and direct case-level linkage were not feasible. Both FAERS and VigiAccess are spontaneous reporting systems and are subject to underreporting, selective reporting, duplicate reporting, missing information, notoriety effects, and indication-related confounding. Moreover, neither database provides a reliable denominator representing the total number of exposed patients. Therefore, the analyses were intended to identify disproportional reporting patterns rather than estimate incidence or establish causal associations between resmetirom and individual adverse events.

### 2.2 Data processing, drug exposure definition, and adverse event coding

FAERS data were standardized and cleaned before analysis. Duplicate case reports were removed according to the FDA-recommended deduplication strategy. For records sharing the same CASEID, the report with the most recent FDA receipt date was retained; when multiple records shared the same CASEID and receipt date, the record with the highest PRIMARYID was retained. Drug names were standardized by harmonizing generic and brand names, capitalization, spelling variants, and abbreviations. Reports containing either “resmetirom” or its brand name “REZDIFFRA” were identified, and the primary analysis was restricted to reports in which resmetirom was designated as the primary suspect (PS) drug. When multiple outcome codes were recorded for the same PRIMARYID, outcomes were classified hierarchically according to a predefined seriousness hierarchy, and only the most serious outcome was retained for descriptive analysis. The predefined hierarchy was death (DE) > life-threatening (LT) > hospitalization (HO) > disability (DS) > congenital anomaly (CA) > required intervention (RI) > other serious outcome (OT). Reports without a recorded serious outcome were classified as non-serious. This approach generated mutually exclusive report-level outcome categories.

Adverse events were coded according to the Medical Dictionary for Regulatory Activities (MedDRA) terminology system (Brown, 2004). Preferred terms (PTs) served as the primary analytical level for detecting signals of disproportionality. In contrast, system organ classes (SOCs) were used to summarize organ-system distributions and to perform SOC-level analyses. For disproportionality analyses, each resmetirom-PT combination constituted a drug–event analytical pair. For report-level demographic, seriousness, outcome, and time-to-onset analyses, unique FAERS reports identified by PRIMARYID were used as the statistical unit to avoid repeated counting of multiple adverse-event terms within the same report.

For temporal analyses, treatment start dates were obtained from the THER table and adverse-event onset dates from the relevant report fields. Clinical outcomes were derived from the OUTC table. Only reports with valid and internally consistent information required for the respective analyses were included; missing or unavailable information was retained as missing in descriptive analyses and was not imputed.

### 2.3 Clinical characteristics, signal detection, seriousness analysis, and concomitant medication analysis

Demographic and clinical characteristics extracted from FAERS included sex, body weight, reporter type, serious outcomes, fatal outcomes, and reporting year (Hu et al., 2025). Serious outcomes were identified according to FAERS outcome criteria, including death, hospitalization, life-threatening events, disability, congenital anomaly, and other medically important outcomes. Missing demographic information was retained as “missing” or “unknown” in descriptive analyses and was not imputed.

Disproportionality analysis was performed using four complementary signal-detection methods: the reporting odds ratio (ROR), proportional reporting ratio (PRR), bayesian confidence propagation neural network (BCPNN), and multi-item gamma poisson shrinker (MGPS) (van Puijenbroek et al., 2002). For each resmetirom-PT pair, a 2 × 2 contingency table was constructed. The Information Component (IC) was used as the signal metric for BCPNN, whereas the Empirical Bayes Geometric Mean (EBGM) was used as the signal metric for MGPS. A positive ROR signal was defined as a ≥3 and the lower limit of the 95% confidence interval >1; a positive PRR signal as a ≥3, PRR ≥2, and χ^2^ ≥ 4; a positive MGPS signal as a ≥3 and EBGM05 > 2; and a positive BCPNN signal as a ≥3 and IC025 > 0. Detailed formulas and signal-detection criteria are provided in Supplementary Table S1. PTs that simultaneously met the predefined positive criteria for all four methods were defined as robust signals. Label expectedness of the robust PT signals was initially assessed against the current United States. Prescribing Information for resmetirom. PTs not directly listed as corresponding adverse reactions were classified as unlabelled signals. The 16 FDA-unlabelled PTs were further verified against the current European Summary of Product Characteristics (SmPC) for Rezdiffra to assess whether these events were listed as corresponding adverse reactions in the European product information (European Medicines Agency, 2026).

To further characterize the clinical relevance of detected signals, report seriousness and clinical outcomes were evaluated at the unique-report level using PRIMARYID as the statistical unit. For PT-level seriousness analysis, duplicate PTs within the same report were removed, and each unique report-PT pair was linked to the corresponding serious or non-serious report status. For each PT, a 2 × 2 contingency table was constructed to compare the presence or absence of the PT across serious and non-serious reports. Fisher’s exact test was used to estimate odds ratios (ORs), corresponding 95% confidence intervals (CIs), and P values. P values were adjusted for multiple testing using the Benjamini–Hochberg false discovery rate (FDR) procedure, and FDR <0.05 was considered statistically significant. Signal profiles were subsequently summarized at both the SOC and PT levels according to report frequency, disproportionality estimates, confidence intervals, seriousness, and statistical precision. To further explore clinically relevant concomitant medications, a descriptive co-medication analysis was performed using the FAERS DRUG table. Drug names were standardized, and selected medications with established or clinically relevant interactions with resmetirom according to the current prescribing information—including atorvastatin, rosuvastatin, pravastatin, simvastatin, clopidogrel, and gemfibrozil—were identified. Each medication was counted once per unique PRIMARYID, and ROLE_COD was retained to distinguish concomitant (C), secondary suspect (SS), and interacting (I) drugs. Because reliable information on dose, treatment duration, temporal overlap, and dechallenge/rechallenge was unavailable, this analysis was descriptive and was not used to estimate formal drug–drug interaction signals.

### 2.4 Time-to-onset and weibull analysis

Time-to-onset (TTO) analysis was restricted to FAERS reports with valid and complete treatment initiation and adverse event onset dates. TTO was defined as the interval, in days, between the reported initiation of resmetirom and the reported onset of the adverse event. Reports with missing, incomplete, implausible, or negative temporal intervals were excluded. TTO was summarized using the median and interquartile range (IQR) and further categorized into predefined onset intervals of 0–30, 31–60, 61–90, 91–180, and >180 days for descriptive analysis. Weibull distribution modeling was used to characterize the temporal distribution of reported adverse event onset times (Sauzet et al., 2013). The scale parameter(α) and shape parameter(β) were estimated together with 95% confidence intervals. An early-failure pattern was defined when the upper limit of the 95% CI for β was <1, whereas a wear-out pattern was defined when the lower limit of the 95% CI was >1. When the 95% CI included 1, the temporal distribution was considered to show no clear departure from β ≈ 1 (Kinoshita et al., 2020).

In addition to the overall analysis, SOC-stratified TTO and Weibull analyses were performed to explore potential heterogeneity in temporal reporting patterns across organ-system categories. Each report contributed at most one TTO observation to a given SOC. SOC-specific estimates were interpreted with caution when based on a limited number of observations. Because TTO analyses were restricted to reports with complete temporal information, the resulting estimates were considered descriptive of the subset of reports that were temporally evaluable rather than representative of all resmetirom reports.

### 2.5 Cross-database consistency assessment using VigiAccess

VigiAccess was used as an external reference source for a descriptive cross-database assessment of the consistency of FAERS-derived signals. VigiAccess data were retrieved through 30 May 2026. Because VigiAccess provides aggregated rather than individual-level case data, the comparison was restricted to PT overlap, SOC-level distributions, PT-level report-count correlation, and relative differences in reporting counts between the two databases. Robust PT signals identified in FAERS were matched with corresponding adverse-event terms reported in VigiAccess, and overlapping PTs were subsequently mapped to their respective SOCs. Spearman’s rank correlation analysis was used to assess the monotonic relationship between PT-level report counts in the two databases. A descriptive Bland–Altman-style plot and the VigiAccess-to-FAERS report-count ratio were additionally used to visualize cross-database differences in reporting patterns. Because the two databases differed in reporting coverage, data structure, and cutoff dates, these analyses were not intended to demonstrate formal statistical agreement or equivalence between the databases. Concordant signals were interpreted as evidence of reproducibility in reporting patterns across two spontaneous-reporting sources rather than as independent external validation or confirmation of causality. Database-specific signals were interpreted with caution, taking into account report frequency, clinical plausibility, product-label information, underlying disease characteristics, and the known limitations of spontaneous reporting systems. Because the publicly accessible VigiAccess data included a small number of reports predating United States. approval and could not be aligned exactly with the quarter-based FAERS observation window, cross-database comparisons were interpreted descriptively rather than as direct time-matched comparisons.

### 2.6 Statistical analysis and ethics

All data processing, statistical analyses, and visualizations were performed using R software version 4.6.0. Categorical variables were summarized as frequencies and percentages. Continuous variables were summarized as means ± standard deviations when approximately normally distributed and as medians with interquartile ranges (IQRs) otherwise. For disproportionality analyses, Bonferroni-adjusted P values were additionally calculated to account for multiple PT-level comparisons; however, Bonferroni significance was not incorporated into the predefined four-method definition of a robust signal. For PT-level comparisons between serious and non-serious reports, Fisher’s exact test was used to estimate ORs, 95% CIs, and P values, and multiple testing was controlled using the Benjamini–Hochberg FDR procedure. Spearman’s rank correlation coefficient was used to assess the association between overlapping PT-level report counts in FAERS and VigiAccess.

Given the high proportions of missing data on sex and body weight, sensitivity analyses were conducted to assess whether missingness in demographics was associated with systematic differences in report characteristics. At the unique-report level, reports with available versus missing sex information and those with available versus missing body-weight information were compared separately. Serious-report status and fatal outcome were compared using Fisher’s exact test, whereas reporter type and reporting year were compared using Pearson’s chi-square test or Fisher’s exact test, as appropriate according to cell counts. Reporter types were grouped into consumer, healthcare professional (including health professional, physician, and pharmacist), or missing to reduce sparse cells. Missing demographic data were not imputed. These sensitivity analyses were considered exploratory, and no additional multiplicity adjustment was applied.

All statistical tests were two-sided. Statistical significance was defined as P < 0.05 for unadjusted analyses and FDR <0.05 for the PT-level seriousness analysis. FAERS and VigiAccess contain publicly accessible, de-identified pharmacovigilance data. No identifiable personal information was accessed or analyzed. Therefore, given the nature of the data used in this study, institutional ethics approval and informed consent were not required.

## 3 Results

### 3.1 Data screening and report inclusion

FAERS reports from 2024Q1, the quarter in which resmetirom received initial United States marketing approval, through 2026Q1 were included in the primary pharmacovigilance analysis. The original FAERS DRUG, DEMO, and REAC tables contained 75,069,409, 24,389,966, and 60,665,903 records, respectively. After data cleaning, deduplication removal, linkage across FAERS tables using PRIMARYID and CASEID, and restriction to reports in which resmetirom was designated as the primary suspect drug, 1,409 unique resmetirom-related reports were retained for analysis. In VigiAccess, 1,417 resmetirom-related reports were retrieved through 30 May 2026. VigiAccess data were used for descriptive cross-database consistency assessment rather than independent external validation. The overall process of data extraction, deduplication, report selection, signal detection, and cross-database comparison is shown in Figure 1.

**FIGURE 1:**
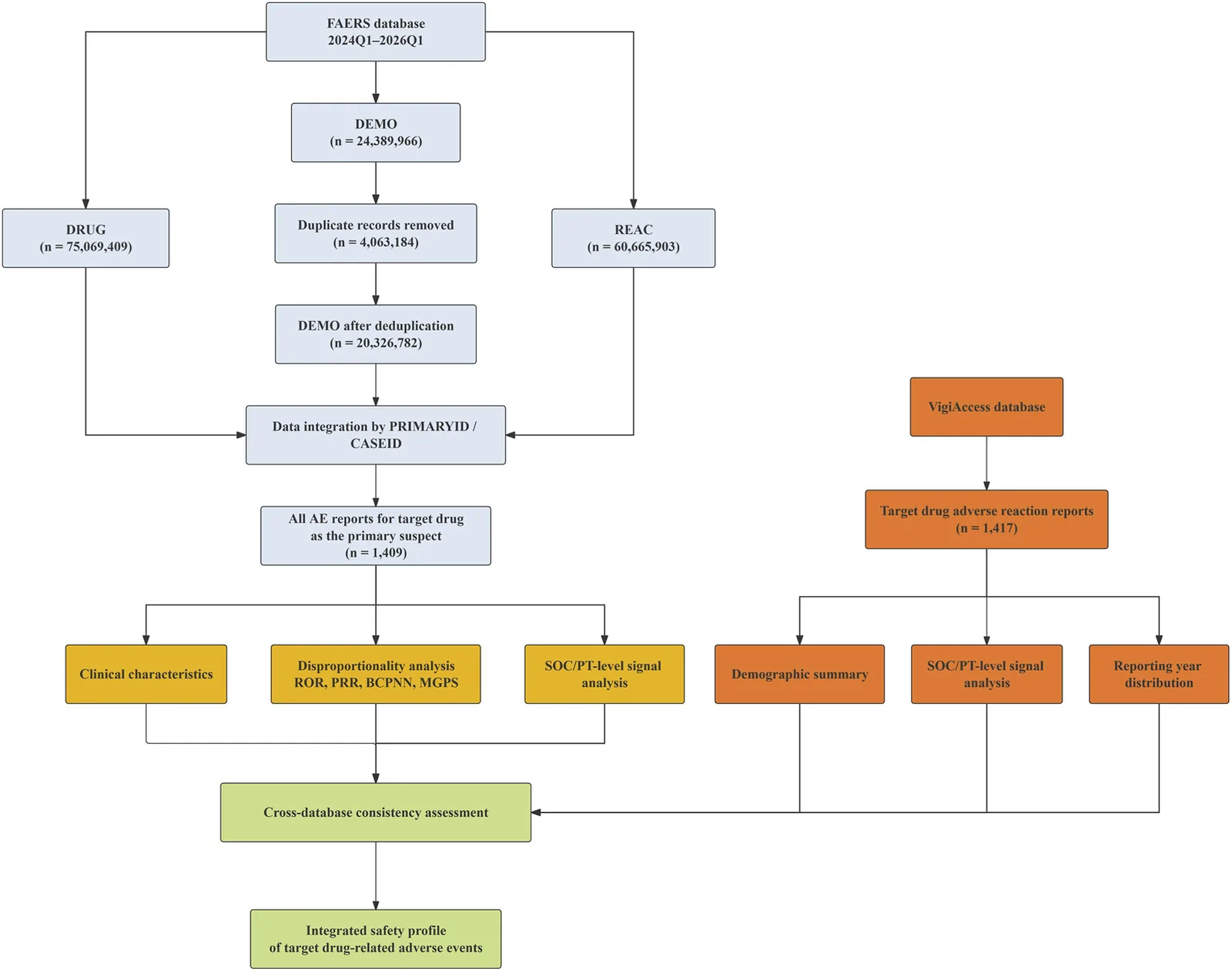
Study workflow for the pharmacovigilance assessment of resmetirom-associated adverse events using the FAERS and VigiAccess databases. The workflow illustrates data extraction, deduplication, identification of Resmetirom-related reports, detection of disproportionality signals using four algorithms (ROR, PRR, BCPNN, and MGPS), SOC/PT-level analyses, and cross-database consistency assessment using VigiAccess.

### 3.2 Demographic and reporting characteristics in FAERS and VigiAccess

A total of 1,409 FAERS reports and 1,417 VigiAccess reports were included. Therefore, differences in reporting-year distributions were interpreted descriptively rather than as direct indicators of reporting intensity or incidence. Demographic information was frequently missing in both databases. In FAERS, sex was unavailable in 1,352 reports (96.0%) and body weight in 1,358 reports (96.4%). Among reports with available reporter information, consumers, healthcare professionals, and physicians represented the major reporter categories. Given the substantial missingness in sex and body-weight information, sensitivity analyses were performed to compare reports with available versus missing demographic data. Reports with available sex information had a significantly higher proportion of serious reports than those with missing sex information (61.4% vs. 28.4%, P < 0.001). In contrast, the proportion of fatal reports did not differ significantly (3.5% vs. 4.7%, P = 1.000). Reporter-type distributions also differed between the two groups (P = 0.035), whereas reporting-year distributions were similar (P = 0.191). Likewise, reports with available body weight information were more frequently classified as serious than those with missing weight information (49.0% vs. 29.0%, P = 0.004), whereas no significant difference in fatal outcomes was observed (0.0% vs. 4.8%, P = 0.168). Reporter-type distributions differed significantly according to weight-data availability (P < 0.001), whereas the difference in reporting-year distribution did not reach statistical significance (P = 0.058) (Supplementary Table S6).

At the report level, 419 FAERS reports (29.7%) were classified as serious and 990 (70.3%) as non-serious. Death was reported in 65 cases (4.6%), hospitalization in 69 (4.9%), life-threatening events in 5 (0.4%), and disability in 5 (0.4%). Reports were predominantly submitted in 2025 in both databases. In VigiAccess, 38.0% of reports were recorded in 2026 compared with 22.1% in FAERS; however, this difference should be interpreted in the context of the later VigiAccess data cutoff rather than as evidence of superior reporting timeliness (Table 1).

**TABLE 1:** Demographic and reporting characteristics of Resmetirom-associated adverse event reports in the FAERS and VigiAccess databases.

Variable	FAERS database (n = 1,409)	VigiAccess database (n = 1,417)
Gender		
Female	32 (2.3%)	50 (3.5%)
Male	25 (1.8%)	28 (2.0%)
Missing	1,352 (96.0%)	1,339 (94.5%)
Weight		
<50 kg	-	-
≥50 and <100 kg	32 (2.3%)	-
>100 kg	19 (1.3%)	-
Missing	1,358 (96.4%)	-
Reporter type	​	​
Health professional	411 (29.2%)	-
Consumer	554 (39.3%)	-
Physician	265 (18.8%)	-
Pharmacist	28 (2.0%)	-
Missing	151 (10.7%)	-
Outcome		
Death	65 (4.6%)	-
Disability	5 (0.4%)	-
Hospitalization	69 (4.9%)	-
Life-threatening	5 (0.4%)	-
Required intervention	3 (0.2%)	-
Other serious outcome	272 (19.3%)	-
No recorded serious outcome	990 (70.3%)	-
Serious report		
No	990 (70.3%)	-
Yes	419 (29.7%)	-
Fatal report		
No	1,344 (95.4%)	-
Yes	65 (4.6%)	-
Year		
2022	-	1 (0.1%)
2023	-	1 (0.1%)
2024	250 (17.7%)	125 (8.8%)
2025	848 (60.2%)	752 (53.1%)
2026	311 (22.1%)	538 (38.00%)

Data are presented as n (%). Percentages were calculated using the total number of reports in each database as the denominator. VigiAccess data for 2026 were available through 30 May 2026. When multiple outcome codes were reported for the same PRIMARYID, outcomes were classified according to a predefined seriousness hierarchy, and only the most serious outcome was retained. Therefore, report-level outcome categories were mutually exclusive. -indicates that data were unavailable or not extracted from VigiAccess for the corresponding characteristic.

### 3.3 SOC-level distribution of adverse events

In FAERS, resmetirom-related adverse event reports were distributed across multiple system organ classes (SOCs), with gastrointestinal disorders representing the largest proportion (24.64%). This was followed by investigations (17.85%), general disorders and administration site conditions (13.30%), skin and subcutaneous tissue disorders (9.92%), hepatobiliary disorders (7.47%), and nervous system disorders (6.22%) (Figure 2A). Overall, the SOC distribution indicated that the reported safety profile of resmetirom was predominantly characterized by gastrointestinal events, laboratory abnormalities, general disorders, skin-related events, and hepatobiliary abnormalities.

**FIGURE 2:**
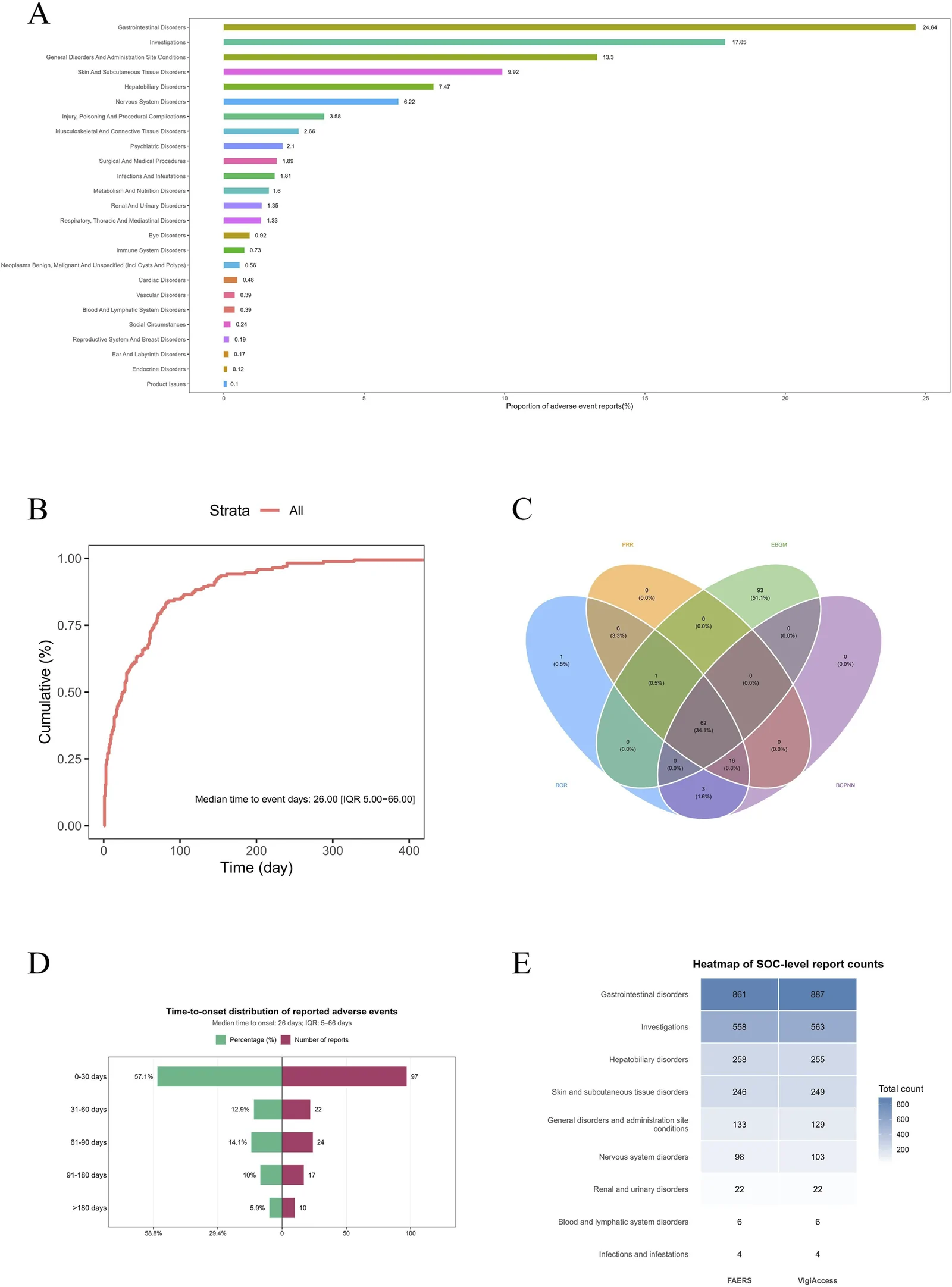
Integrated characterization of resmetirom-associated adverse event reports. **(A)** Distribution of adverse event reports across system organ classes (SOCs). **(B)** Empirical cumulative distribution of reported time-to-onset (TTO). **(C)** Venn diagram showing overlap among signals identified by ROR, PRR, BCPNN, and MGPS. **(D)** Distribution of reported onset-time intervals. **(E)** Heatmap comparing SOC-level report counts between FAERS and VigiAccess.

### 3.4 PT-level adverse event signal detection

Using the four predefined signal-detection methods (ROR, PRR, BCPNN, and MGPS), 62 PTs simultaneously met all four positivity criteria and were therefore classified as robust disproportionality signals (Figure 2C). Complete PT-level disproportionality analysis results are provided in Supplementary Table S2. These signals were distributed across nine SOCs, with investigations, hepatobiliary disorders, and gastrointestinal disorders contributing the largest numbers of signal-positive PTs. When considered by cumulative report count, gastrointestinal disorders accounted for the greatest reporting burden, followed by investigations, hepatobiliary disorders, and skin and subcutaneous tissue disorders (Table 2).

**TABLE 2:** Robust preferred term (PT) signals meeting all four disproportionality criteria for resmetirom in FAERS.

SOC	PT	Case	ROR (95% CI)	PRR (χ^2^)	EBGM(EBGM05)	IC(IC025)	P value	Statistical test	Bonferroni-adjusted P value
Skin and subcutaneous tissue disorders	Pruritus	216	8.69 (7.58–9.97)	8.29 (1,392.91)	8.29 (7.23)	3.05 (2.8)	P < 0.001	Chi-square	P < 0.001
Skin and subcutaneous tissue disorders	Yellow skin	30	97.26 (67.83–139.44)	96.56 (2,818.7)	95.93 (66.91)	6.58 (4.04)	P < 0.001	Fisher’s exact test	P < 0.001
Renal and urinary disorders	Chromaturia*	22	14.39 (9.46–21.88)	14.32 (272.4)	14.31 (9.41)	3.84 (2.58)	P < 0.001	Fisher’s exact test	P < 0.001
Nervous system disorders	Dizziness	87	2.67 (2.16–3.3)	2.64 (89.11)	2.64 (2.13)	1.4 (1.06)	P < 0.001	Chi-square	P < 0.001
Nervous system disorders	Brain fog*	11	12.31 (6.81–22.25)	12.28 (113.87)	12.27 (6.79)	3.62 (1.83)	P < 0.001	Fisher’s exact test	P < 0.001
Investigations	Alanine aminotransferase increased	127	31.94 (26.76–38.11)	30.99 (3,681.22)	30.92 (25.91)	4.95 (4.39)	P < 0.001	Fisher’s exact test	P < 0.001
Investigations	Aspartate aminotransferase increased	122	35.41 (29.57–42.41)	34.39 (3,949.8)	34.32 (28.65)	5.1 (4.49)	P < 0.001	Fisher’s exact test	P < 0.001
Investigations	Weight decreased*	82	4.5 (3.62–5.6)	4.43 (218.75)	4.43 (3.56)	2.15 (1.77)	P < 0.001	Chi-square	P < 0.001
Investigations	Hepatic enzyme increased	61	13.77 (10.69–17.73)	13.58 (710.95)	13.57 (10.54)	3.76 (3.13)	P < 0.001	Fisher’s exact test	P < 0.001
Investigations	Blood bilirubin increased	55	30.84 (23.63–40.26)	30.45 (1,563.88)	30.39 (23.28)	4.93 (3.93)	P < 0.001	Fisher’s exact test	P < 0.001
Investigations	Blood alkaline phosphatase increased	29	17.41 (12.08–25.1)	17.3 (444.97)	17.28 (11.99)	4.11 (2.96)	P < 0.001	Fisher’s exact test	P < 0.001
Investigations	Liver function test increased	15	10.76 (6.48–17.87)	10.73 (132.23)	10.72 (6.45)	3.42 (2.01)	P < 0.001	Fisher’s exact test	P < 0.001
Investigations	Hepatic fibrosis marker increased	9	7,783.58 (3,467.61–17471.4)	7,766.64 (45,691.94)	5,078.53 (2,262.51)	12.31 (2.25)	P < 0.001	Fisher’s exact test	P < 0.001
Investigations	Hepatic enzyme decreased	7	242.04 (114.62–511.12)	241.63 (1,650.26)	237.73 (112.58)	7.89 (1.93)	P < 0.001	Fisher’s exact test	P < 0.001
Investigations	Blood thyroid stimulating hormone decreased*	6	19.73 (8.85–43.96)	19.7 (106.37)	19.68 (8.83)	4.3 (1.33)	P < 0.001	Fisher’s exact test	P < 0.001
Investigations	Alanine aminotransferase decreased	4	84.39 (31.57–225.6)	84.31 (327.42)	83.84 (31.36)	6.39 (0.96)	P < 0.001	Fisher’s exact test	P < 0.001
Investigations	Aspartate aminotransferase decreased	4	115.4 (43.12–308.8)	115.29 (449.65)	114.4 (42.75)	6.84 (0.97)	P < 0.001	Fisher’s exact test	P < 0.001
Hepatobiliary disorders	Hepatic cirrhosis	69	56.95 (44.87–72.28)	56.02 (3,715.43)	55.81 (43.97)	5.8 (4.62)	P < 0.001	Fisher’s exact test	P < 0.001
Hepatobiliary disorders	Ocular icterus	29	102.27 (70.89–147.54)	101.56 (2,867.9)	100.87 (69.92)	6.66 (4.01)	P < 0.001	Fisher’s exact test	P < 0.001
Hepatobiliary disorders	Jaundice	26	14.51 (9.86–21.34)	14.42 (324.58)	14.41 (9.8)	3.85 (2.71)	P < 0.001	Fisher’s exact test	P < 0.001
Hepatobiliary disorders	Metabolic dysfunction-associated steatohepatitis	23	286.33 (189.31–433.07)	284.74 (6,379.42)	279.34 (184.69)	8.13 (3.87)	P < 0.001	Fisher’s exact test	P < 0.001
Hepatobiliary disorders	Hepatic pain	17	60.27 (37.39–97.13)	60.02 (982.72)	59.78 (37.09)	5.9 (3.13)	P < 0.001	Fisher’s exact test	P < 0.001
Hepatobiliary disorders	Hepatic failure	14	6.99 (4.14–11.81)	6.97 (71.58)	6.97 (4.12)	2.8 (1.57)	P < 0.001	Fisher’s exact test	P < 0.001
Hepatobiliary disorders	Liver injury	12	7.62 (4.32–13.43)	7.6 (68.78)	7.6 (4.31)	2.93 (1.53)	P < 0.001	Fisher’s exact test	P < 0.001
Hepatobiliary disorders	Hepatic fibrosis	11	48.73 (26.94–88.15)	48.61 (511.23)	48.45 (26.78)	5.6 (2.45)	P < 0.001	Fisher’s exact test	P < 0.001
Hepatobiliary disorders	Suspected drug-induced liver injury	4	362.58 (134.39–978.22)	362.23 (1,406.21)	353.53 (131.03)	8.47 (1)	P < 0.001	Fisher’s exact test	P < 0.001
General disorders and administration site conditions	Disease progression	37	4.76 (3.44–6.58)	4.73 (108.85)	4.72 (3.42)	2.24 (1.63)	P < 0.001	Chi-square	P < 0.001
General disorders and administration site conditions	No adverse event	35	2.96 (2.12–4.13)	2.94 (44.97)	2.94 (2.11)	1.56 (1)	P < 0.001	Chi-square	P < 0.001
General disorders and administration site conditions	Unevaluable event	27	5.4 (3.7–7.88)	5.37 (96.09)	5.37 (3.68)	2.42 (1.67)	P < 0.001	Chi-square	P < 0.001
General disorders and administration site conditions	Adverse event	22	3.61 (2.38–5.49)	3.6 (41.31)	3.6 (2.37)	1.85 (1.09)	P < 0.001	Chi-square	P < 0.001
Gastrointestinal disorders	Diarrhoea	244	6.05 (5.32–6.89)	5.75 (967.38)	5.75 (5.05)	2.52 (2.31)	P < 0.001	Chi-square	P < 0.001
Gastrointestinal disorders	Nausea	218	4.36 (3.8–4.99)	4.18 (534.09)	4.18 (3.65)	2.06 (1.84)	P < 0.001	Chi-square	P < 0.001
Gastrointestinal disorders	Abdominal pain upper	96	7.22 (5.9–8.84)	7.08 (502.26)	7.07 (5.78)	2.82 (2.44)	P < 0.001	Chi-square	P < 0.001
Gastrointestinal disorders	Vomiting	87	2.84 (2.3–3.52)	2.81 (101.88)	2.81 (2.27)	1.49 (1.15)	P < 0.001	Chi-square	P < 0.001
Gastrointestinal disorders	Abdominal pain	62	4.07 (3.16–5.23)	4.02 (141.23)	4.02 (3.13)	2.01 (1.57)	P < 0.001	Chi-square	P < 0.001
Gastrointestinal disorders	Constipation	58	4.12 (3.18–5.34)	4.08 (135.08)	4.08 (3.14)	2.03 (1.57)	P < 0.001	Chi-square	P < 0.001
Gastrointestinal disorders	Abdominal discomfort	42	3.71 (2.74–5.03)	3.69 (82.43)	3.69 (2.72)	1.88 (1.35)	P < 0.001	Chi-square	P < 0.001
Gastrointestinal disorders	Flatulence	22	5.92 (3.9–9.01)	5.9 (89.53)	5.9 (3.88)	2.56 (1.68)	P < 0.001	Fisher’s exact test	P < 0.001
Gastrointestinal disorders	Faeces pale*	6	40.88 (18.34–91.16)	40.83 (232.47)	40.72 (18.26)	5.35 (1.52)	P < 0.001	Fisher’s exact test	P < 0.001
Investigations	High density lipoprotein decreased*	3	13.4 (4.32–41.58)	13.39 (34.36)	13.38 (4.31)	3.74 (0.26)	P < 0.01	Fisher’s exact test	1.000
Infections and infestations	Urosepsis*	4	6.52 (2.45–17.4)	6.52 (18.68)	6.52 (2.44)	2.7 (0.34)	P < 0.01	Fisher’s exact test	1.000
Hepatobiliary disorders	Hepatic cyst*	3	13.33 (4.29–41.37)	13.32 (34.16)	13.31 (4.29)	3.73 (0.26)	P < 0.01	Fisher’s exact test	1.000
Gastrointestinal disorders	Abnormal faeces	4	7.38 (2.77–19.68)	7.37 (22.03)	7.37 (2.76)	2.88 (0.4)	P < 0.01	Fisher’s exact test	1.000
Gastrointestinal disorders	Gastrointestinal pain*	4	5.52 (2.07–14.71)	5.51 (14.78)	5.51 (2.07)	2.46 (0.24)	P < 0.01	Fisher’s exact test	1.000
Hepatobiliary disorders	Cholecystitis	5	6.36 (2.65–15.3)	6.36 (22.56)	6.35 (2.64)	2.67 (0.57)	P < 0.01	Fisher’s exact test	0.885
Hepatobiliary disorders	Autoimmune hepatitis	4	8.74 (3.28–23.3)	8.73 (27.37)	8.73 (3.27)	3.13 (0.49)	P < 0.01	Fisher’s exact test	0.861
Investigations	Transaminases increased	7	4.75 (2.26–9.97)	4.74 (20.69)	4.74 (2.26)	2.25 (0.67)	P < 0.001	Fisher’s exact test	0.567
Hepatobiliary disorders	Hepatomegaly*	5	7.57 (3.15–18.2)	7.56 (28.45)	7.56 (3.14)	2.92 (0.67)	P < 0.001	Fisher’s exact test	0.412
Investigations	Serum ferritin increased*	4	11.42 (4.28–30.47)	11.41 (37.98)	11.41 (4.28)	3.51 (0.6)	P < 0.001	Fisher’s exact test	0.321
Investigations	Blood testosterone increased*	3	20.97 (6.76–65.11)	20.96 (56.94)	20.93 (6.74)	4.39 (0.36)	P < 0.001	Fisher’s exact test	0.297
Investigations	Blood cholesterol decreased	3	24.28 (7.82–75.38)	24.26 (66.8)	24.22 (7.8)	4.6 (0.39)	P < 0.001	Fisher’s exact test	0.194
Investigations	International normalised ratio increased*	9	4.65 (2.42–8.94)	4.64 (25.72)	4.64 (2.41)	2.21 (0.85)	P < 0.001	Fisher’s exact test	0.127
General disorders and administration site conditions	Therapeutic response unexpected	12	3.65 (2.07–6.44)	3.64 (23.04)	3.64 (2.07)	1.87 (0.8)	P < 0.001	Fisher’s exact test	0.112
Blood and lymphatic system disorders	Splenomegaly*	6	7.7 (3.46–17.15)	7.69 (34.91)	7.69 (3.45)	2.94 (0.88)	P < 0.001	Fisher’s exact test	0.109
Hepatobiliary disorders	Hepatotoxicity	8	5.49 (2.74–10.98)	5.48 (29.29)	5.48 (2.74)	2.45 (0.91)	P < 0.001	Fisher’s exact test	0.095
Investigations	Hepatic enzyme abnormal	5	15.18 (6.31–36.52)	15.17 (66.11)	15.15 (6.3)	3.92 (0.99)	P < 0.001	Fisher’s exact test	P < 0.05
Hepatobiliary disorders	Liver disorder	13	4.43 (2.57–7.64)	4.42 (34.46)	4.42 (2.57)	2.14 (1.06)	P < 0.001	Fisher’s exact test	P < 0.01
Investigations	Thyroxine decreased	3	71.5 (22.99–222.39)	71.45 (207.37)	71.1 (22.86)	6.15 (0.49)	P < 0.001	Fisher’s exact test	P < 0.01
Gastrointestinal disorders	Anal incontinence*	8	8.78 (4.39–17.57)	8.76 (55.01)	8.76 (4.38)	3.13 (1.27)	P < 0.001	Fisher’s exact test	P < 0.01
Hepatobiliary disorders	Cholelithiasis	12	5.51 (3.13–9.71)	5.49 (44.13)	5.49 (3.12)	2.46 (1.23)	P < 0.001	Fisher’s exact test	P < 0.01
Gastrointestinal disorders	Faeces discoloured*	10	6.97 (3.74–12.96)	6.95 (50.95)	6.95 (3.74)	2.8 (1.3)	P < 0.001	Fisher’s exact test	P < 0.01
Hepatobiliary disorders	Hepatitis alcoholic	3	136.78 (43.86–426.51)	136.68 (400.33)	135.43 (43.43)	7.08 (0.52)	P < 0.001	Fisher’s exact test	P < 0.01

* Indicates robust PT signals not directly listed as corresponding adverse reactions in the current U.S. Prescribing Information. These PTs were additionally verified against the current EMA SmPC and were likewise not listed as corresponding adverse reactions.

The most frequently reported robust PT signals were diarrhoea (n = 244; ROR = 6.05, 95% CI: 5.32–6.89), nausea (n = 218; ROR = 4.36, 95% CI: 3.80–4.99), pruritus (n = 216; ROR = 8.69, 95% CI: 7.58–9.97), alanine aminotransferase increased (n = 127; ROR = 31.94, 95% CI: 26.76–38.11), aspartate aminotransferase increased (n = 122; ROR = 35.41, 95% CI: 29.57–42.41). Other frequently reported signals included abdominal pain upper, dizziness, vomiting, weight decreased, and hepatic cirrhosis. Gastrointestinal signals were predominantly characterized by relatively high counts, including diarrhoea, nausea, abdominal pain, vomiting, constipation, abdominal discomfort and flatulence (Table 2; Supplementary Figure S1).

Investigation-related signals primarily involved hepatic biochemical abnormalities, including alanine aminotransferase increased, aspartate aminotransferase increased, hepatic enzymes increased, blood bilirubin increased, blood alkaline phosphatase increased, and liver function test increased. Several lower-frequency investigation PTs also showed high estimates of disproportionality. In particular, the hepatic fibrosis marker increased in only nine cases but yielded an exceptionally high ROR (7,783.58, 95% CI:3,467.61–17,471.40). Given the small number of reports and the extremely wide effect estimate, this finding should be interpreted as a sparse-data signal rather than evidence of a correspondingly large clinical risk.

Hepatobiliary signals included hepatic cirrhosis, ocular icterus, jaundice, metabolic dysfunction-associated steatohepatitis, hepatic pain, hepatic failure, liver disorder, liver injury, cholelithiasis, hepatic fibrosis, hepatotoxicity, and cholecystitis. Hepatic cirrhosis was reported in 69 cases (ROR = 56.95, 95% CI:44.87–72.28), metabolic dysfunction-associated steatohepatitis in 23 cases (ROR = 286.33, 95% CI: 189.31–433.07), and suspected drug-induced liver injury in four cases (ROR = 362.58, 95% CI: 134.39–978.22). Because several of these PTs overlap clinically with the underlying MASH disease process, their disproportionality estimates should be interpreted cautiously in the context of potential indication-related confounding.

Among other PT-level signals, pruritus (n = 216; ROR = 8.69, 95% CI: 7.58–9.97) and yellow skin (n = 30; ROR = 97.26, 95% CI: 67.83–139.44) were prominent skin-related signals. Dizziness (n = 87; ROR = 2.67, 95% CI: 2.16–3.30) and brain fog (n = 11; ROR = 12.31, 95% CI: 6.81–22.25)were identified within nervous system disorders, whereas chromaturia (n = 22; ROR = 14.39, 95% CI: 9.46–21.88) was identified within renal and urinary disorders. Low-frequency signals, particularly those based on fewer than 20 reports, were considered exploratory due to greater statistical uncertainty.

PT-level seriousness analysis identified 19 PTs that differed significantly between serious and non-serious reports after Benjamini–Hochberg FDR correction. Common gastrointestinal events, including diarrhoea and nausea, were more frequently represented among non-serious reports. In contrast, several hepatobiliary and disease-progression-related PTs, including hepatic fibrosis, metabolic dysfunction-associated steatohepatitis, disease progression, cholelithiasis, hepatic cirrhosis, hepatic failure, liver injury, drug-induced liver injury, and hepatotoxicity, were disproportionately represented among serious reports (Supplementary Table S4).

Label expectedness assessment identified 16 robust PT signals that were not directly listed as corresponding adverse reactions in the current United States. Prescribing Information. These included weight decreased, chromaturia, brain fog, and several other lower-frequency PTs. Further verification against the current European Summary of Product Characteristics (SmPC) for Rezdiffra showed that these 16 PTs were likewise not listed as corresponding adverse reactions. Accordingly, these findings were classified as previously unlabelled disproportionality signals rather than newly established adverse drug reactions (Figure 3A).

**FIGURE 3:**
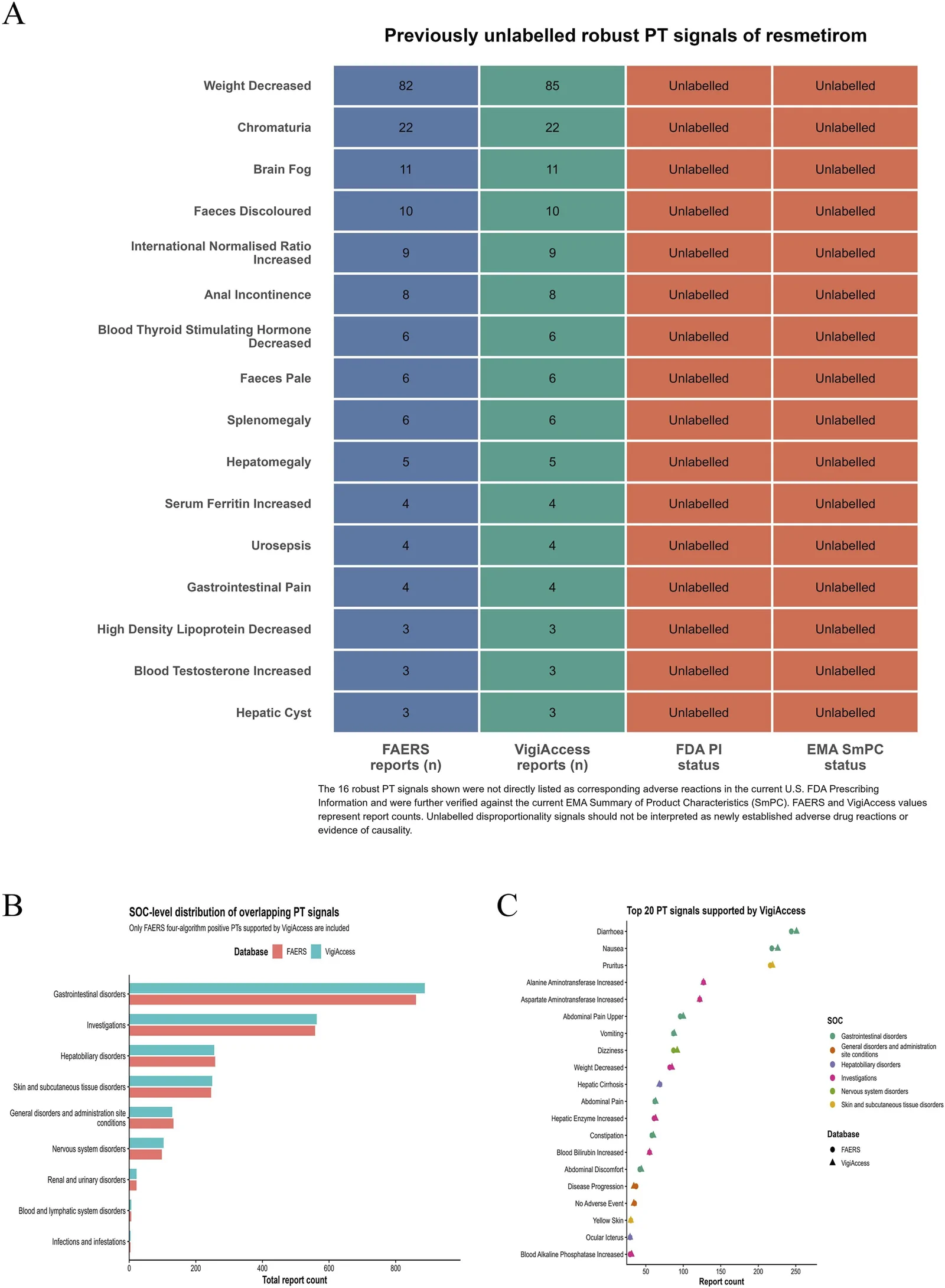
Regulatory expectedness and cross-database comparison of resmetirom-associated adverse event signals. **(A)** Regulatory expectedness assessment of the 16 robust PT signals not directly listed in the current United States Prescribing Information and further verified against the EMA Summary of Product Characteristics (SmPC). FAERS and VigiAccess report counts are shown together with the FDA and EMA label status. **(B)** SOC-level distribution of overlapping PT reports between FAERS and VigiAccess. **(C)** Top 20 overlapping PTs according to report counts in the two databases.

Descriptive analysis of clinically relevant concomitant medications showed that 205 of 1,409 FAERS reports (14.5%) included at least one of the four statins highlighted in the current prescribing information. Atorvastatin was the most frequently identified (n = 111, 7.9%), followed by rosuvastatin (n = 68, 4.8%), pravastatin (n = 16, 1.1%), and simvastatin (n = 12, 0.9%). Because individual reports could contain more than one target statin, medication-specific counts were not mutually exclusive. Clopidogrel, a moderate CYP2C8 inhibitor, was recorded in 11 reports (0.8%), whereas gemfibrozil, a strong CYP2C8 inhibitor, was recorded in three reports (0.2%). Nine reports (0.6%) contained both a target statin and clopidogrel, including six with atorvastatin and three with rosuvastatin. No report contained both a target statin and gemfibrozil. Most of these medications were coded as concomitant drugs rather than suspect drugs (Supplementary Table S7). These findings represent descriptive co-reporting patterns and should not be interpreted as evidence of causal drug–drug interactions.

### 3.5 Time-to-onset and weibull distribution analysis

Among the 1,409 FAERS reports, 170 (12.1%) contained complete and valid information on resmetirom initiation and adverse event onset dates and were included in the overall time-to-onset (TTO) analysis. The median TTO was 26 days (IQR, 5–66 days) (Table 3). The empirical cumulative distribution of reported onset times showed that reports accumulated predominantly during the early treatment period, followed by a progressively slower increase over time (Figure 2B). However, only 170 of 1,409 reports (12.1%) had complete temporal information, and the resulting TTO estimates may therefore be affected by selection bias.

**TABLE 3:** Overall time-to-onset characteristics and Weibull distribution parameters of resmetirom-associated adverse event reports.

Number of cases	TTO, median (IQR)	Weibull scale parameter, α (95% CI)	Weibull shape parameter, β (95% CI)	Failure type
170	26 (5–66)	40.52 (31.59–49.44)	0.72 (0.64–0.80)	Early failure

Abbreviations: TTO, time to onset; IQR, interquartile range; CI, confidence interval.

α represents the scale parameter of the Weibull distribution, and β represents the shape parameter.

An early-failure pattern was defined when the upper limit of the 95% CI for β was <1; a wear-out pattern was defined when the lower limit of the 95% CI for β was >1. When the 95% CI included 1, no clear departure from β = 1 was considered evident.

When categorized by onset interval, 97 reports (57.1%) occurred within 0–30 days after treatment initiation, followed by 22 (12.9%) at 31–60 days, 24 (14.1%) at 61–90 days, 17 (10.0%) at 91–180 days, and 10 (5.9%) after 180 days (Figure 2D). Thus, among reports with available temporal information, more than half of the reported adverse events occurred within the first 30 days after initiation of resmetirom.

Overall, Weibull modeling yielded a scale parameter(α) of 40.52 (95% CI: 31.59–49.44) and a shape parameter(β) of 0.72 (95% CI: 0.64–0.80) (Table 3). Because the upper limit of the 95% CI for β was below 1, the overall reported onset-time distribution was consistent with an early failure-type pattern. This finding indicates that reported onset times were more concentrated during the earlier treatment period; however, it should not be interpreted as evidence of a higher incidence or absolute clinical risk during this period.

SOC-stratified analyses further demonstrated heterogeneous temporal reporting patterns (Supplementary Table S5). Gastrointestinal disorders had the earliest median onset of 9 days and showed an early-failure pattern (β = 0.606, 95% CI, 0.543–0.686). Skin and subcutaneous tissue disorders (median, 15.5 days; β = 0.719, 95% CI, 0.612–0.870), general disorders and administration site conditions (median, 40 days; β = 0.701, 95% CI, 0.591–0.860), and nervous system disorders (median, 10 days; β = 0.693, 95% CI, 0.579–0.862) also showed early-failure patterns. In contrast, hepatobiliary disorders had a later median onset of 70 days, and the 95% CI for β included 1 (β = 0.937, 95% CI, 0.741–1.272), indicating no clear departure from β = 1. SOCs with limited numbers of temporally evaluable reports were considered exploratory.

### 3.6 Cross-database consistency assessment between FAERS and VigiAccess

To assess cross-database consistency, the 62 robust PT signals identified in FAERS were matched with corresponding adverse-event reports in VigiAccess (Supplementary Table S3). Overall, the two databases showed broadly similar reporting patterns at both the SOC and PT levels. At the SOC level, FAERS and VigiAccess showed broadly similar reporting patterns, with gastrointestinal disorders, investigations, hepatobiliary disorders, and skin and subcutaneous tissue disorders representing the predominant categories in both databases (Figures 2E and 3B).

At the PT level, frequently reported events identified in FAERS were also represented in VigiAccess, including diarrhoea, nausea, pruritus, alanine aminotransferase increased, aspartate aminotransferase increased, abdominal pain upper, vomiting, dizziness, weight decreased, hepatic cirrhosis, abdominal pain, hepatic enzymes increased, and constipation (Figure 3C). Spearman correlation analysis demonstrated a strong positive association between PT-level report counts in the two databases (ρ = 0.998, P < 0.001), indicating a high degree of similarity in the relative reporting patterns of overlapping PTs (Figure 4A).

**FIGURE 4:**
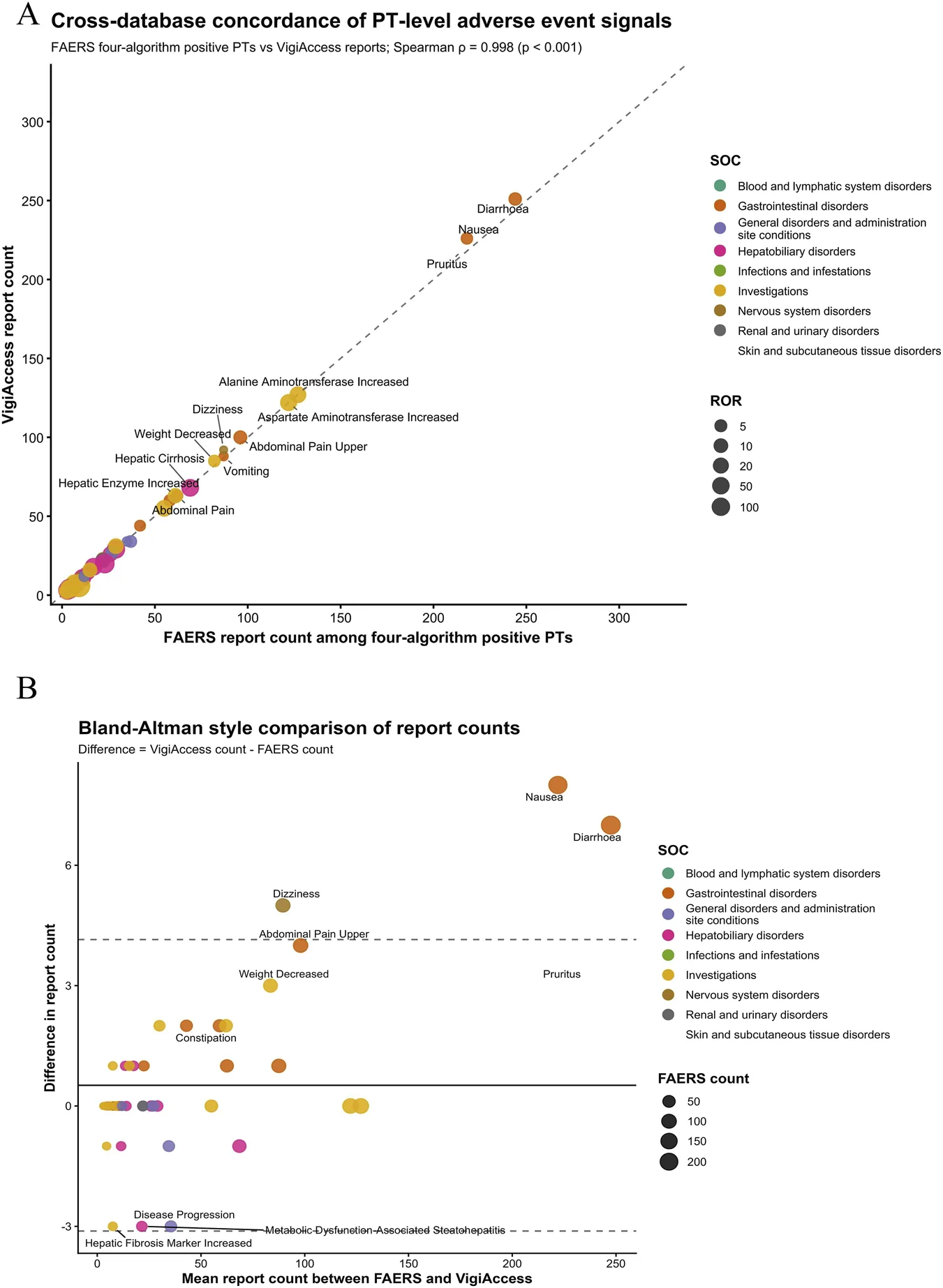
Cross-database comparison of PT-level report counts between FAERS and VigiAccess. **(A)** Correlation analysis of PT-level report counts. **(B)** Descriptive Bland–Altman-style comparison of differences in PT-level report counts between the two databases.

A descriptive Bland-Altman-style comparison further illustrated the distribution of differences in PT-level report counts between FAERS and VigiAccess (Figure 4B). The VigiAccess-to-FAERS report-count ratio additionally showed that the magnitude of cross-database differences varied across individual PTs (Supplementary Figure S2). These comparisons were interpreted descriptively because FAERS and VigiAccess differ in reporting coverage, data structure, and cutoff dates, and potential overlap of individual reports between the two spontaneous-reporting systems could not be excluded. Taken together, the cross-database analysis demonstrated concordant reporting patterns for several major gastrointestinal, investigation-related, hepatobiliary, and skin-related PTs. However, this concordance should be regarded as supportive pharmacovigilance evidence rather than independent external validation or confirmation of a causal association.

## 4 Discussion

Resmetirom is the first selective thyroid hormone receptor-β (THR-β) agonist approved for the treatment of noncirrhotic MASH with moderate-to-advanced liver fibrosis, representing an important advance in the pharmacological management of MASH (Kannt et al., 2021; Keam, 2024). With the increasing global burden of obesity and metabolic syndrome, NASH has become an important contributor to progressive liver fibrosis, cirrhosis and hepatocellular carcinoma (Motta et al., 2023; Yopp and Choti, 2015). In this post-marketing pharmacovigilance analysis, 62 robust PT signals across nine SOCs were identified using four complementary signal-detection methods. Gastrointestinal events accounted for the largest reporting burden, whereas investigation-related and hepatobiliary PTs included several signals with high estimates of disproportionality. Seriousness analysis further demonstrated a distinct pattern: common gastrointestinal events such as diarrhoea and nausea were predominantly represented among non-serious reports, whereas several hepatobiliary and disease-progression-related PTs were enriched among serious reports. Among reports with evaluable onset information, adverse events were frequently reported early after treatment initiation, although SOC-stratified analyses revealed substantial heterogeneity in temporal patterns. Cross-database comparison with VigiAccess showed broadly concordant reporting patterns, providing supportive pharmacovigilance evidence rather than independent external validation.

Gastrointestinal adverse events represented the most prominent and consistently reported safety findings in this study (Harrison et al., 2023; Yopp and Choti, 2015). Diarrhoea, nausea, vomiting, and abdominal pain upper showed clear disproportionality signals, with diarrhoea and nausea ranking among the most frequently reported robust PTs. These findings are broadly consistent with the gastrointestinal safety profile reported in clinical studies and the current prescribing information (Harrison et al., 2023; Shuaibi et al., 2025). In the present analysis, gastrointestinal disorders also showed an early temporal pattern, with a median reported onset of 9 days and an early-failure Weibull distribution. In addition, commonly reported gastrointestinal PTs were predominantly observed in non-serious reports, suggesting they may primarily reflect treatment tolerability rather than severe clinical outcomes. Although resmetirom is designed to selectively activate hepatic THR-β, thyroid hormone signaling has systemic metabolic effects, and modulation of gastrointestinal function may contribute to some of these symptoms (Kudaravalli et al., 2025; Saponaro et al., 2020). Nevertheless, the precise mechanisms underlying resmetirom-associated gastrointestinal events remain incompletely understood. Clinically, attention to gastrointestinal tolerability during the early treatment period, together with patient education, dietary adjustment, and appropriate symptomatic management, may help improve treatment adherence (Au et al., 2024).

Laboratory abnormalities and hepatobiliary signals were more clinically complex and require cautious interpretation. Signals involving alanine aminotransferase increased, aspartate aminotransferase increased, blood bilirubin increased, and blood alkaline phosphatase increased were detected, together with hepatobiliary PTs such as hepatic cirrhosis, hepatic fibrosis, hepatic failure, liver injury, hepatotoxicity, and suspected drug-induced liver injury (Shuaibi et al., 2025). Unlike the predominantly non-serious gastrointestinal PTs, several hepatobiliary and disease-progression-related PTs were more frequently represented among serious reports. However, these findings are particularly susceptible to indication-related confounding and protopathic bias. Patients receiving resmetirom already have MASH with moderate-to-advanced fibrosis and therefore remain at risk of persistent hepatic inflammation, fibrosis progression, cirrhosis, and abnormal liver biochemistry. Consequently, some liver-related reports may represent progression or clinical manifestations of the underlying disease rather than direct drug toxicity (Do et al., 2025).

The temporal findings also support this interpretation. Although the overall TTO analysis showed an early-failure pattern, SOC-stratified analyses demonstrated substantial heterogeneity. Hepatobiliary disorders had a relatively later median reported onset than gastrointestinal disorders and showed no clear departure from β = 1. Therefore, the overall early-onset pattern should not be generalized to all resmetirom-related reports, particularly hepatobiliary events. At the same time, THR-β activation substantially alters hepatic lipid metabolism and may also influence pathways related to bile acid and cholesterol homeostasis (Saponaro et al., 2020; Musso et al., 2013; Walenbergh and Shiri-Sverdlov, 2015). Importantly, the current prescribing information includes warnings about hepatotoxicity and gallbladder-related adverse reactions, such as cholelithiasis and cholecystitis (U.S. Food and Drug Administration, 2026). Thus, liver-related abnormalities occurring during treatment should be interpreted in conjunction with baseline liver disease severity, temporal relationships, biochemical patterns, and alternative causes.

From a clinical perspective, evaluation of suspected hepatobiliary toxicity should include relevant liver biochemical parameters, including ALT, AST, alkaline phosphatase, and total bilirubin, together with assessment for competing etiologies when appropriate. Where sufficient laboratory data are available, the biochemical pattern of suspected drug-induced liver injury may be further characterized using the R ratio, while marked aminotransferase elevation accompanied by hyperbilirubinemia should prompt assessment for a Hy’s law-type pattern and systematic exclusion of alternative causes (Fontana et al., 2023). Symptoms such as right upper quadrant pain, marked fatigue, dark urine, jaundice, or scleral icterus warrant further evaluation. In patients with suspected gallbladder-related complications, hepatobiliary imaging may also be appropriate. Particularly large estimates of disproportionality based on small numbers of reports should be interpreted with caution. For example, hepatic fibrosis marker increased showed an extremely high ROR despite being reported in only nine cases, indicating substantial disproportionality but also considerable sparse-data uncertainty. Such findings should therefore be regarded as exploratory pharmacovigilance signals rather than estimates of the magnitude of clinical risk. Of particular interest, 16 robust PT signals were not directly listed as corresponding adverse reactions in either the current United States. Prescribing Information or the EMA SmPC (U.S. Food and Drug Administration, 2026; European Medicines Agency, 2026). These findings should be interpreted as previously unlabelled signals of disproportionality rather than as confirmed novel adverse drug reactions. Although the concordant absence of these PTs from both regulatory documents may highlight areas requiring further surveillance, spontaneous reporting data alone cannot determine whether these signals represent true drug-related effects. Some may reflect variability in reporting, underlying disease manifestations, concomitant conditions, or residual confounding. Further confirmation using larger longitudinal cohorts and denominator-based data is warranted.

The robust signals of pruritus and yellow skin also warrant contextual interpretation. Pruritus is a recognized adverse reaction associated with resmetirom, but in patients with chronic liver disease, it may also occur in association with cholestatic or hepatobiliary dysfunction (Asal et al., 2021). Accordingly, pruritus should not automatically be interpreted as either an isolated dermatological event or evidence of liver injury. When accompanied by jaundice, dark urine, or abnormalities in bilirubin, alkaline phosphatase, or other liver biochemical parameters, additional assessment for hepatobiliary dysfunction may be warranted. This is particularly relevant because yellow skin, ocular icterus, jaundice, and several bilirubin-related PTs were also identified in the present analysis.

Drug-drug interactions are particularly relevant in patients with MASH because cardiometabolic comorbidities and polypharmacy are common in this population, with statins, antidiabetic agents, and other long-term therapies frequently co-administered. Hepatic uptake transporters, including OATP1B1, are involved in the disposition of resmetirom and contribute to its liver-directed pharmacologic profile (Hönes et al., 2022). Concomitant administration of resmetirom with statins that depend in part on OATP-mediated hepatic uptake, including atorvastatin and rosuvastatin, may increase statin exposure (U.S. Food and Drug Administration, 2026; Balasubramanian and Maideen, 2021). Clinically relevant interactions also involve CYP2C8: clopidogrel, a moderate CYP2C8 inhibitor, was identified in 11 reports, whereas gemfibrozil, a strong CYP2C8 inhibitor, was identified in three reports. Nine reports contained both a target statin and clopidogrel. According to the current prescribing information, concomitant use with strong CYP2C8 inhibitors, such as gemfibrozil, is not recommended, whereas a resmetirom dose reduction is recommended with moderate CYP2C8 inhibitors, such as clopidogrel (U.S. Food and Drug Administration, 2026). Accordingly, medication reconciliation, adherence to recommended dose limitations, and appropriate monitoring are important during concomitant therapy. However, because FAERS does not reliably capture dose, duration, temporal overlap, adherence, or dechallenge/rechallenge information, these findings should be regarded as descriptive co-medication patterns rather than formal drug–drug interaction signals.

Cross-database comparison with VigiAccess demonstrated broadly concordant reporting patterns for several major gastrointestinal, investigation-related, hepatobiliary, and skin-related PTs. PT-level report counts showed a strong positive correlation between FAERS and VigiAccess (Spearman ρ = 0.998). However, this high correlation should not be interpreted as independent external validation of the FAERS-derived signals. Both databases are based on spontaneous reporting and therefore share important limitations, including underreporting, selective reporting, missing data, and stimulated reporting. Moreover, the potential overlap of individual reports between FAERS and the global reports available in VigiAccess cannot be ruled out. Accordingly, the observed concordance should be regarded as supportive evidence of similar reporting patterns across the two data sources rather than confirmation of causality or proof that database-specific reporting bias has been eliminated. Mild or self-limited symptoms, delayed-onset events, subclinical laboratory abnormalities, and events that are difficult to distinguish from underlying MASH progression may be systematically underreported in both databases. Consequently, cross-database concordance may preferentially reflect more salient or more readily attributable events.

From a practical monitoring perspective, baseline assessment should include liver biochemistry and medication reconciliation, with particular attention to CYP2C8 inhibitors and concomitant statin therapy. Gastrointestinal tolerability should be assessed particularly during the early treatment period, whereas periodic liver chemistry monitoring should continue throughout treatment. New jaundice, dark urine, right upper quadrant pain, or substantial liver-test abnormalities should prompt further evaluation for hepatotoxicity and alternative hepatic causes. At the same time, symptoms suggestive of gallbladder disease warrant appropriate imaging and clinical follow-up. These recommendations represent a pragmatic monitoring framework rather than a validated risk-prediction algorithm.

This study has several limitations. First, both FAERS and VigiAccess are spontaneous reporting systems and are subject to underreporting, selective reporting, missing information, and reporting stimulation effects (Sun et al., 2026). Because reliable exposure denominators are unavailable, disproportionality analyses identify reporting signals rather than incidence rates, relative risks, or causal associations (Potter et al., 2025). Second, resmetirom has only recently entered clinical practice, and the number of accumulated post-marketing reports remains relatively limited; some findings may therefore be influenced by stimulated reporting or notoriety effects associated with a newly approved drug. Third, demographic information was highly incomplete, particularly for sex and body weight. Sensitivity analyses indicated systematic differences between reports with available demographic information and those with missing information: reports with available sex or body weight information were more frequently classified as serious. They showed differences in reporter-type distribution compared with reports lacking these data. Consequently, findings for demographic subgroups should be interpreted with substantial caution, and the present data do not support reliable identification of sex- or weight-specific high-risk populations. Nevertheless, the primary disproportionality analyses were based on the full set of eligible resmetirom reports and did not depend on the availability of sex or body-weight information. In addition, only 170 of the 1,409 FAERS reports (12.1%) contained complete and valid information for TTO analysis; therefore, the resulting temporal estimates may be affected by selection bias.

## 5 Conclusion

This study identified robust post-marketing disproportionality signals for resmetirom, predominantly involving gastrointestinal, investigation-related, hepatobiliary, and skin-related PTs. Gastrointestinal events tended to be reported earlier and were largely non-serious, whereas several hepatobiliary PTs were more frequently associated with serious reports and should be interpreted with caution due to potential confounding by underlying MASH. FAERS and VigiAccess showed broadly concordant reporting patterns, providing supportive but non-causal evidence in pharmacovigilance. These findings highlight the importance of monitoring gastrointestinal tolerability, liver-related abnormalities, gallbladder symptoms, and clinically relevant drug-drug interactions during resmetirom treatment.

## Data Availability

The original contributions presented in the study are included in the article/Supplementary Material, further inquiries can be directed to the corresponding author.

## Supplementary material

SUPPLEMENTARY FIGURE S1 Forest plot of PT-level adverse event signals associated with resmetirom in FAERS. Points represent reporting odds ratios (RORs), horizontal lines represent 95% confidence intervals (CIs), and the dashed vertical line indicates ROR = 1. PTs are grouped by system organ class (SOC).

SUPPLEMENTARY FIGURE S2 Relative reporting patterns of overlapping PTs between FAERS and VigiAccess. The x-axis shows the VigiAccess-to-FAERS report-count ratio; ratios >1 indicate relatively higher report counts in VigiAccess, and ratios <1 indicate relatively higher report counts in FAERS. Point size represents the FAERS report count, and color indicates SOC.

SUPPLEMENTARY TABLE S1 Algorithms, formulas, and signal-detection criteria used in pharmacovigilance analyses. ROR, reporting odds ratio; PRR, proportional reporting ratio; BCPNN, Bayesian Confidence Propagation Neural Network; MGPS, Multi-item Gamma Poisson Shrinker; IC, information component; IC025, lower 95% credibility interval of the information component; EBGM, empirical Bayes geometric mean; EBGM05, lower 95% credibility limit of EBGM; CI, confidence interval.

SUPPLEMENTARY TABLE S2 Complete list of PT-level disproportionality analysis results for Resmetirom in the FAERS database. FAERS, FDA Adverse Event Reporting System; PT, preferred term; SOC, system organ class; ROR, reporting odds ratio; PRR, proportional reporting ratio; IC, information component; EBGM, empirical Bayes geometric mean; CI, confidence interval.

SUPPLEMENTARY TABLE S3 Cross-database comparison of FAERS-derived robust PT signals with corresponding reports in VigiAccess. FAERS, FDA Adverse Event Reporting System; PT, preferred term; SOC, system organ class.

SUPPLEMENTARY TABLE S4 PT-level comparison between serious and non-serious resmetirom reports in FAERS. FAERS, FDA Adverse Event Reporting System; PT, preferred term; OR, odds ratio; CI, confidence interval; FDR, false discovery rate.

SUPPLEMENTARY TABLE S5 SOC-stratified time-to-onset and Weibull distribution analysis of resmetirom-related adverse event reports in FAERS. FAERS, FDA Adverse Event Reporting System; SOC, system organ class; TTO, time to onset; IQR, interquartile range; CI, confidence interval; α, Weibull scale parameter; β, Weibull shape parameter.

SUPPLEMENTARY TABLE S6 Sensitivity analysis comparing report characteristics according to the availability of demographic information in FAERS. OR, odds ratio; CI, confidence interval. Odds ratios and 95% CIs were estimated for binary outcomes using Fisher’s exact test. Reporter type and reporting year were compared using Pearson’s chi-square test or Fisher’s exact test, as appropriate, according to cell counts. Healthcare professionals included health professionals, physicians and pharmacists. No imputation was performed for missing demographic data.

SUPPLEMENTARY TABLE S7 Descriptive analysis of selected clinically relevant concomitant medications in resmetirom reports from FAERS. C, concomitant; SS, secondary suspect; I, interacting. Each medication was counted once per unique PRIMARYID. ROLE_COD was used to characterize the reported role of each co-medication. This analysis was descriptive; the presence of a concomitant medication does not establish a drug-drug interaction or causal relationship with the reported adverse event. Because individual reports could contain more than one target statin, medication-specific counts were not mutually exclusive.
